# A Case Report of Tongue Lymphoepithelial Carcinoma with a Histological Diagnostic Dilemma

**DOI:** 10.3390/diagnostics11061039

**Published:** 2021-06-04

**Authors:** Daisuke Takeda, Manabu Shigeoka, Tenyu Sugano, Nanae Yatagai, Takumi Hasegawa, Masaya Akashi

**Affiliations:** 1 Division of Oral and Maxillofacial Surgery, Department of Surgery Related, Kobe University Graduate School of Medicine, 7-5-2 Kusunoki-cho, Chuo-ku, Kobe 650-0017, Japan; dsktkd@med.kobe-u.ac.jp (D.T.); y.pipi.1339@gmail.com (N.Y.); hasetaku@med.kobe-u.ac.jp (T.H.); akashim@med.kobe-u.ac.jp (M.A.); 2 Division of Pathology, Department of Pathology, Kobe University Graduate School of Medicine, 7-5-1 Kusunoki-cho, Chuo-ku, Kobe 650-0017, Japan; 3 Division of Diagnostic Pathology, Department of Pathology, Kobe University Graduate School of Medicine, 7-5-2 Kusunoki-cho, Chuo-ku, Kobe 650-0017, Japan; suga1ten@gmail.com

**Keywords:** tongue cancer, lymphoepithelial carcinoma, EBER negative, histopathological diagnosis, oral cavity

## Abstract

Most head and neck lymphoepithelial carcinomas (LECs) arise in the nasopharynx and harbor Epstein–Barr virus (EBV). LEC is also a rare subtype of the oral squamous cell carcinoma (SCC). Morphologically, LEC is defined as resembling non-keratinizing nasopharyngeal carcinoma, undifferentiated subtype. The histological features and pathogenesis of oral LEC are not established. We describe a case of tongue LEC with histopathological diagnostic difficulties. A 72-year-old Japanese female presented with a whitish change on her left-side tongue. The diagnosis was atypical epithelium; neoplastic change could not be ruled out by a biopsy. Although the lesion was monitored at our hospital per her request, invasive carcinoma was detected 11 months later. Microscopically, conventional SCC was observed with the characteristic features as LEC confined to the deep part of the lesion. We briefly discuss this unusual histological finding and make a novel proposal for distinguishing oral LEC from LECs in other regions based on these histological findings.

## 1. Introduction

Oral squamous cell carcinoma (SCC) is a carcinoma with squamous differentiation arising from the mucosal epithelium [1]. There are architectural and cytological epithelial changes associated with an increased risk of progression to SCC. Unlike mucosal SCC with a high mutational burden due to chronic alcohol or tobacco exposure, virus-associated carcinomas have a distinct pathogenesis [2].

Lymphoepithelial carcinoma (LEC) is a ‘subtype’ of oral SCC [1]. In the World Health Organization (WHO) classification, LEC is defined as an “SCC morphologically similar to non-keratinizing nasopharyngeal carcinoma (NK-NPC), undifferentiated subtype” [1]. LEC is mostly located in the nasopharynx, and NK-NPC has a strong association with Epstein–Barr virus (EBV) [1]. EBV infection is consistently associated with LEC epidemiology and pathogenesis [3], but differences are noted depending on geographic regions and affected sites [4,5,6]. Once infected with EBV, the virus lies latent in the epithelial cells of the oropharynx and salivary glands and in B-lymphocytes and persists as a low-grade active infection throughout life [3].

Oral LEC is extremely rare, and not all LECs are EBV-positive [1]. The histological features of oral LECs and the biologic significance of EBV detection in LECs are not established. Different pathogenesis may be present in oral LEC without EBV detection. We report a case of tongue LEC with a histological diagnostic dilemma.

## 2. Case Presentation

**Figure 1:**
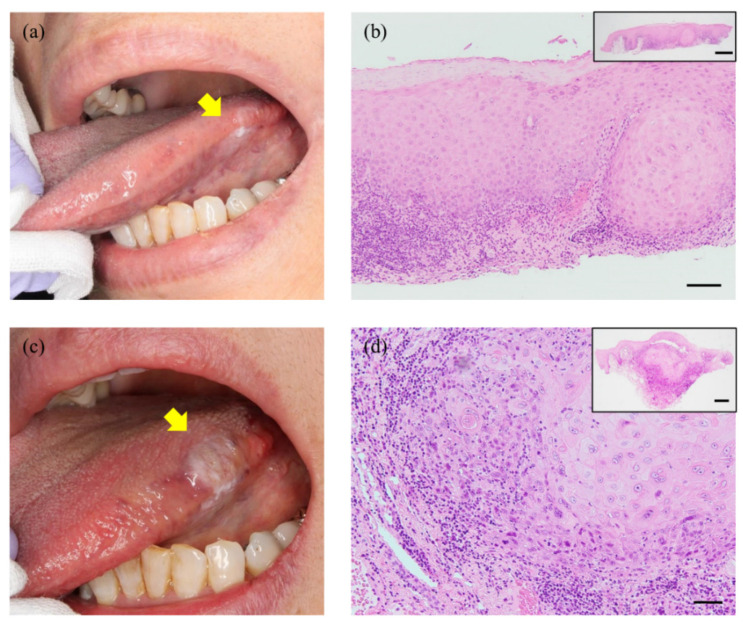
Clinical appearance and histological findings of the patient’s biopsy. (**a**) A whitish lesion was found on the left lingual edge at her first visit (*arrow*). (**b**) This lesion was histologically diagnosed as ‘atypical epithelium that does not rule out neoplastic change’ based on this first biopsy. (**c**) After 11 months of monitoring, a mass with induration was observed (*arrow*). (**d**) A morphological evaluation by second biopsy confirmed invasive SCC with dyskeratosis. Scale bars: 100 µm (**b**), 50 µm (**d**). *Insets* in (**b**,**d**) are lower magnifications of the images (scale bars: 500 µm).

A 72-year-old Japanese female was referred to our hospital for the examination of a whitish lesion on her left tongue. She became aware of its existence with discomfort 5 months prior to this presentation. She had a history of appendicitis and clear cell renal cell carcinoma, but the EBV infection had not been pointed out. Intraoral examination revealed a unilateral white patch with non-detachable at the left lingual edge (Figure 1a). We performed a local biopsy, and the histological diagnosis ‘atypical epithelium that does not rule out neoplastic change’ was obtained (Figure 1b). As she did not desire surgical treatment, we monitored the lesion, and 11 months later, a mass with induration was observed.

The exophytic lesion was ~12 × 6 mm in maximum diameter, and a pale whitish lesion was present in the anterior part (Figure 1c). We performed a second biopsy, and the histological diagnosis of SCC was confirmed (Figure 1d). Contrast-enhanced CT and FDG-PET showed no cervical lymph node metastasis. The clinical stage was thus classified as cT2N0M0 based on the TNM classification (8th edition) of the Union for International Cancer Control [7].

**Figure 2:**
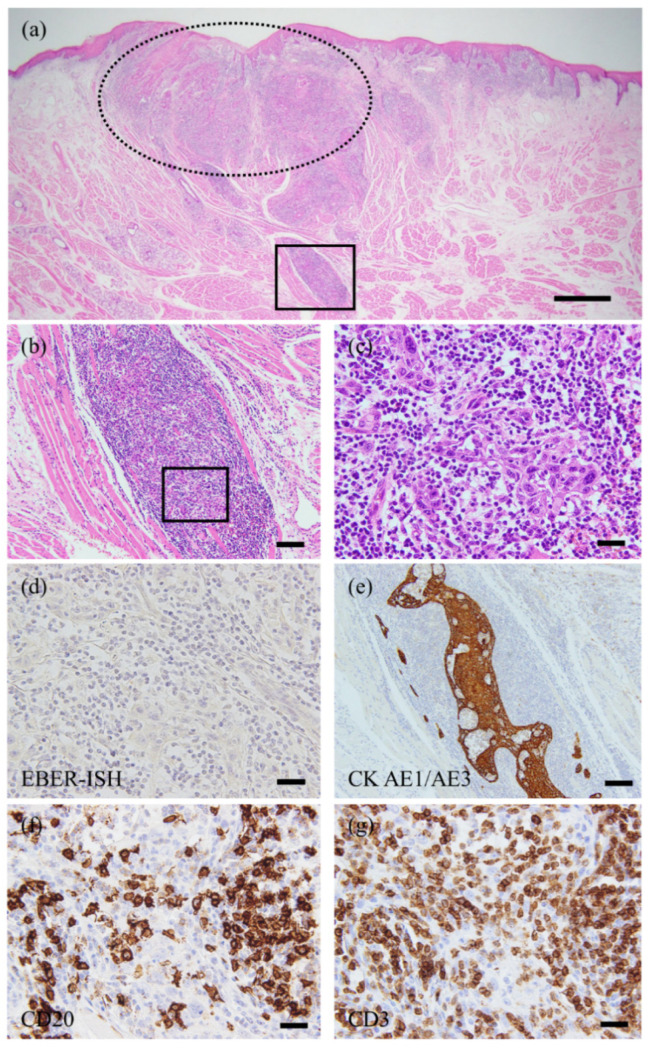
The morphological and immunohistochemical images. (**a**) Low-power image (HE staining). Invasive cancer was detected in resected specimens. The depth of invasion was 4.6 mm. The lesion was classified as pT1cN0M0. *Dotted line* indicates a feature of conventional SCC. (**b**) In the deep area (square A), the observation of the cancer nests was difficult due to lymphoplasmacytic infiltration. Both keratinization and desmoplastic stromal reaction were absent. (**c**) High-power view of the invasive front (square B). The tumor cells have enlarged vesicular nuclei with prominent nucleoli. Nuclear pleomorphism is also increased. (**d**) The tumor cells were negative for EBER-ISH. (**e**) The tumor cells were positive for CK AE1/AE3. (**f**,**g**) The monoclonal reactivities for both a B-cell (CD20) and a T-cell (CD3) marker in infiltrating lymphocytes could not be detected. Scale bars: 1 mm (**a**), 100 µm (**b**,**e**), 20 µm (**c**,**d**,**f**,**g**).

We performed a partial glossectomy with 10-mm tumor-free margins. A microscopic examination was performed using whole-tissue sections. The hematoxylin–eosin (HE)-stained specimens revealed a 4.6-mm-deep moderately differentiated conventional SCC characterized by invasion into the lamina propria with little keratin pearl formation, and cellular and nuclear pleomorphism was observed in whole tissue (Figure 2a). Solid nests with non-keratinization and lymphoplasmacytic cell infiltration at a deep stromal area were observed; the desmoplastic stromal reaction was not remarkable (Figure 2b). These findings are unusual patterns as conventional SCC. At higher magnification, the tumor cells showed large round-to-oval nuclei with hyperchromasia, an increased nuclear-to-cytoplasmic ratio, and prominent nucleoli; the indistinct cell border showed a syncytial appearance (Figure 2c). These findings were thought to be morphologically similar to NK-NPC. The pathologists discussed this histological diagnostic dilemma, LEC was considered in histological differential diagnosis.

In situ hybridization (ISH) was negative for EBV-encoded RNA (EBER) in the tumor cells (Figure 2d). These tumor cells were highlighted by a pan-cytokeratin marker, CK AE1/AE3 (Figure 2e), and tumor-infiltrating lymphocytes (TILs) did not show monoclonal reactivity for a B-cell marker (CD20) or a T-cell marker (CD3) (Figure 2f,g). The diagnosis of LEC (pT1cN0M0, stage I) was confirmed. She showed no evidence of disease at the 1-year follow-up. Written informed consent was obtained from the patient for the publication of this case report.

## 3. Discussion

The microscopic features of oral LEC are not established, due to the limited number of this rare tumor. We have identified only 16 cases of tongue LEC in the English literature [8,9,10], and all 16 cases were at the base of the tongue, and their association with EBV was not described. We have presented a case of LEC occurring at the lingual edge, which is extremely uncommon.

The most peculiar feature of the present case is the overlap of two histological features: conventional SCC and LEC. Although the whole cancer tissue exhibited features of conventional SCC, poorly differentiated malignant epithelial cells accompanied by TILs were observed in the deep area. The desmoplastic reaction has been highlighted as an important stromal reaction in various solid tumors, including oral SCC [11,12,13,14]. Unusually, the desmoplastic reaction was unremarkable at the invasive front in our patient. The lesion thus was finally diagnosed as LEC.

Oral SCCs are commonly well or moderately differentiated, and poorly differentiated cases are much less [1]. It was recently proposed that the characteristics of oral carcinogenesis differ from those of carcinogenesis of the uterine cervix or esophagus because oral SCC retains the maturation and differentiation characteristics of the stratified squamous epithelium [15,16,17]. It is thus reasonable to consider that the characteristics of oral LEC should be distinguished from LECs occurring at other sites. The specific findings of LEC as a ‘*subtype*’ of oral SCC characterized by superficial maturation and differentiation might be likely to appear in the deep area of the tumor tissue. Although we should recognize that only limited conclusions can be drawn from this single case, this histological finding might provide a clue to the differential diagnosis.

Most cases of head and neck LEC harbor EBV [1], but several reports [18,19,20,21] described cases of EBV-negative LEC in oral mucosa and adjacent structures [22]. Consistent with these reports, the ISH results was negative for EBV in our patient. These findings support our hypothesis that the characteristics of LEC in oral mucosa differ from those at other head and neck sites. To clarify what causes these differences, the pathogenesis of EBV-negative LEC should be determined.

Head and neck LECs tend to metastasize to regional lymph nodes, which affects the prognosis [1,9,23,24], and these tumors have high sensitivity to radiotherapy [1,9,23,25]. Our patient*’*s tumor was completely resected, and thus we did not conduct additional radiotherapy. In addition, no cervical lymph nodes suspected of metastasis were present preoperatively, we considered additional prophylactic neck dissection to be overtreatment. Despite the tendency of LECs in the prior reports to metastasize or spread locally, the prognosis remains rather favorable compared to other poorly differentiated epithelial tumors [24]. It was also suggested that patients with EBV-positive malignancies have better survival than those with EBV-negative malignancies, as is the case for oncogenic human papillomavirus-positive oropharyngeal cancer [21]. We plan to longitudinally monitor our patient.

In conclusion, this case report provides three take-home messages for readers. (1) While the tongue LECs in all of the published reports arose at the base of the tongue, this tongue LEC occurred at the lingual edge. (2) The most peculiar feature of the present case is the overlap of histological features of conventional SCC and LEC. The whole cancer tissue indicated conventional SCC, but poorly differentiated malignant epithelial cells and TILs were observed in the deep area. Although we should recognize the weakness of the single case, we would like to stress this histological feature as a promising clue to distinguish oral LEC from LECs at other sites. (3) Consistent with previous reports, in situ hybridization (ISH) was negative for EBV in this patient with oral LEC. These findings support our hypothesis that the characteristics of LEC in oral mucosa differ from those at other head and neck sites. To clarify what caused these differences, the pathogenesis of EBV-negative LEC should be determined in the future.
